# Application of Space Technologies Aimed at Proprioceptive Correction in Terrestrial Medicine in Russia

**DOI:** 10.3389/fphys.2022.921862

**Published:** 2022-06-16

**Authors:** Eugenia Motanova, Maria Bekreneva, Ilya Rukavishnikov, Tatiana A. Shigueva, Alina A. Saveko, Elena S. Tomilovskaya

**Affiliations:** Institute of Biomedical Problems, Russian Academy of Sciences (RAS), Moscow, Russia

**Keywords:** Proprioceptive correction, support afferentation, neurorehabilitation, Korvit, axial loading suit

## Abstract

Space technologies greatly contributed not only to space medicine but also to terrestrial medicine, which actively involves these technologies in everyday practice. Based on the existing countermeasures, and due to similarities of sensorimotor alterations provoked by the weightlessness with various neurological disorders, a lot of work has been dedicated to adaptation and introduction of these countermeasures for rehabilitation of patients. Axial loading suit and mechanical stimulation of the soles’ support zones are used in mitigation of stroke and traumatic brain injury consequences. They are also applied for rehabilitation of children with cerebral palsy. Complex application of these proprioceptive correction methods in neurorehabilitation programs makes it possible to effectively treat neurological patients with severe motor disturbances and significant brain damage.

## 1 Introduction

Biomedical support for manned space missions is comprised by advanced medical achievements. Not only its development upgraded existing medical technologies, but it also led to invention of unique methods and approaches. The major focus of aerospace physiology and medicine revolved around prevention and reduction of space flight associated negative effects, mostly around the effects of microgravity and hypokinesia. All the biomedical data obtained during space flight and ground-based model experiments led to better understanding of physiological characteristics and capabilities of human body in the conditions of real and simulated microgravity. In addition, space biomedical research enlarged existing knowledge of how the body adapts to the conditions of ambient environment and the underlying mechanisms of this adaptation. This helped to create new ergonomic and reliable equipment and techniques that can be used in extreme conditions and confined environment. The importance of these space developments and their customized versions is also in their integration with terrestrial medicine, as they are applied for diagnosis establishment, treatment and prevention of various diseases and rehabilitation therapy (Legostaev, Markov and Sorokin, 2013; Orlov, Belakovskiy and Kussmaul, 2014; Orlov, Kussmaul and Belakovskiy, 2020).

## 2 Space Technologies in Neurorehabilitation

Even though scientific and technological progress simplified human life in general, it also triggered certain problems, such as deprivation of motor activity, which resulted in increasing numbers of people suffering from various disorders of musculoskeletal system. Nowadays hypokinesia is a vexed problem of all age groups and is considered to be among the main causes of various metabolic disorders (metabolic syndrome, obesity, type II diabetes mellitus), diseases of cardiovascular system (ischemic heart disease, atherosclerosis, hypertension), sarcopenia—an age-related decrease in muscle mass and functional capabilities. Besides, there are people with forcedly limited physical activity, such as patients who need to maintain prolonged bed rest and who are especially susceptible to negative influence of hypokinesia factors (Grigoriev, 2001; Popov, 2018). In the past few years scientists have acquired significant amount of data suggesting that hypokinesia and hypokinesia-related changes in the regulatory and metabolic mechanisms intensify the course of pathological processes. Support withdrawal (weightlessness) and a decrease in volume of support stimuli (hypokinesia) strongly reduce slow-twitch (tonic) motor units’ activity. This reduction is always accompanied by muscle atony and absolute muscle strength decline (Kozlovskaya et al., 2007). In fact, the more the percentage of slow-twitch fibers, the higher the decrease of absolute muscle strength. Exposure to microgravity and full elimination of support provokes a condition called hypogravity motor syndrome: short-term exposure results in decreased muscle tone and muscle contractile capabilities (Kozlovskaya, 2018). Along with that, it provokes osteoporosis (demineralization of bone tissue), body ataxia and apraxia, alters body scheme perception and movement biomechanics. It also induces coordination disorders manifested by a sharp decrease in vertical stability, postural synergy system disruption, and changes in the structure of motor acts (Williams et al., 2009).

Due to similarities of sensorimotor alterations provoked by weightlessness and various pathological disorders, a lot of work has been dedicated to adaptation of existing space countermeasures for rehabilitation of patients, suffering from severe motor disturbances, triggered by children cerebral palsy, ischemic stroke, brain injuries and other spinal and cardiovascular pathologies (Kozlovskaya et al., 2006, 2007).

## 3 Axial Loading Suit for Neurorehabilitation

**FIGURE 1:**
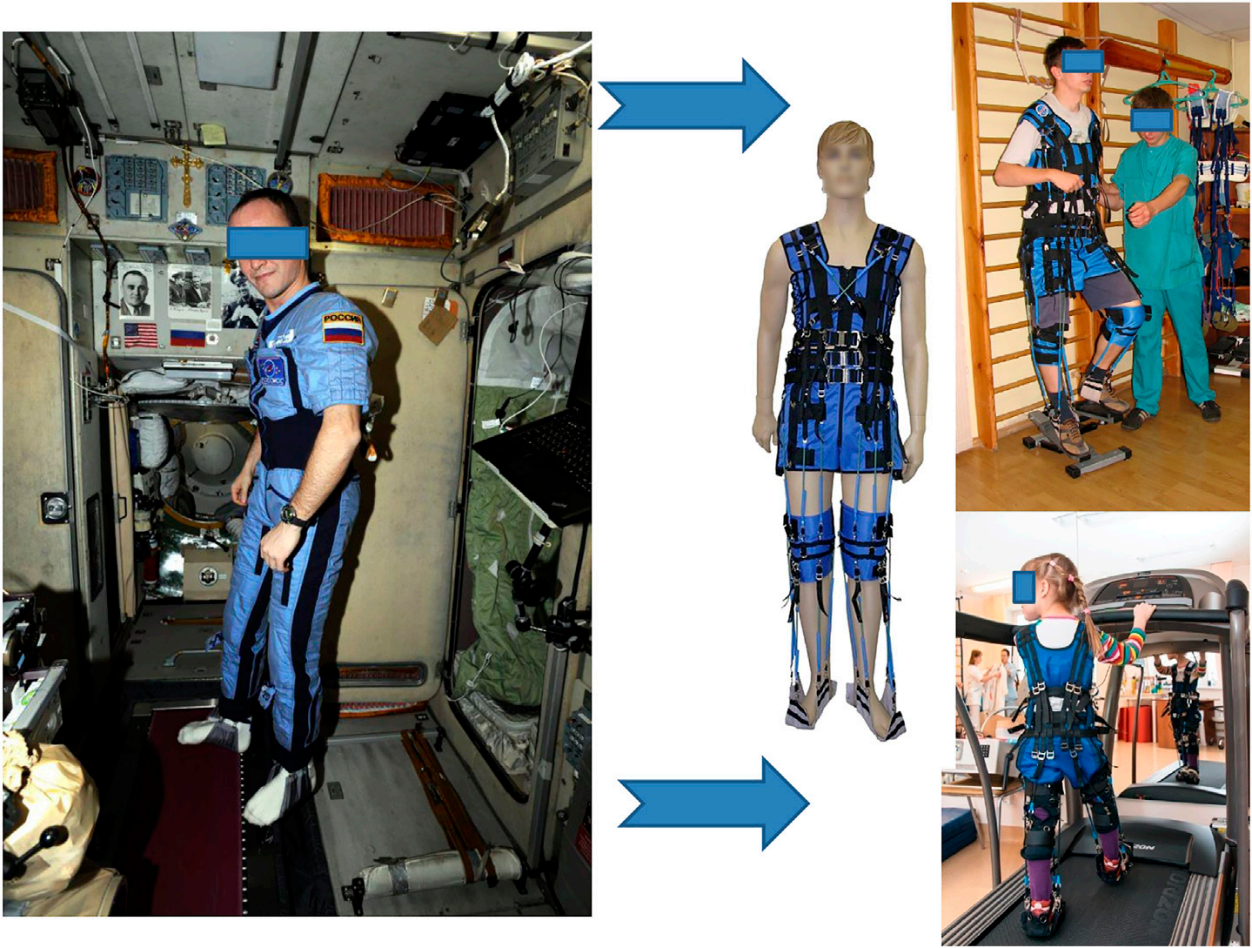
Axial loading suit. On the left—“Penguin” space suit and its application on board the International Space Station; on the right–its clinical version “Regent” (Center of Aerospace Medicine and Technologies, Russia) and its adaptation for rehabilitation of adult patients and children. Photo credit IBMP

While preparing cosmonauts for long-term space flights, the Institute of Biomedical Problems of the Russian Academy of Sciences and “Zvezda” Research and Production Association developed “Penguin” axial loading suit. The main purpose of this suit was to create axial load and compensate the absence of support and proprioceptive afferentation in microgravity and during hypokinesia, thus mitigating negative effects of weightlessness. Action of the suit is associated with direct influence on proprioceptors of muscles and joints and with simultaneous correction of afferent vestibulo-proprioceptive flow on the central structures of motor control brain areas. Motor afferentation has a profound activating effect on human brain: the flow of proprioceptive stimuli changes the functional properties of neurons, transforms them into polymodal neurons and provides them with increased susceptibility to stimuli of various sensory modalities. The suit has a system of elastic loading elements, which are distributed according to the antagonist muscles’ topography. The suit corrects initial posture asymmetries and limits body’s excessive degrees of freedom by creating load on musculoskeletal system and increasing resistance for movement performance (Figure 1) (Yarmanova et al., 2015).

In the early 90s, “Penguin” was modified into “Adeli” medical suit and then used in a dynamic proprioceptive correction method. This method was first used in 1991 as a complex treatment of cerebral spastic infantile paralysis at the Institute of Pediatrics of the Russian Academy of Sciences (Semenova and Antonova, 1998). This method allowed children with severe motor defects to develop walking skills in a shorter period of time, improve vertical stability, posture, functional state of neuromotor apparatus and intellectual functions (Semenova, 1997; Nemkova SA. et al., 2000; Nemkova S. A. et al, 2000). “Regent” medical suit (a “Penguin” suit modification) was used for rehabilitation of patients with motor disorders caused by stroke and traumatic brain injury. Design of the “Regent” suit is different from the above mentioned models, as its elastic loading elements can be removed from the suit and reattached in diverse ways. Such variability in their distribution allows to create multidirectional load on the body (Galanov et al., 2010; Makarova et al., 2012). “Regent” suit is most frequently used for neurorehabilitation nowadays. The complex influence of this suit is defined by a number of factors:

• Axial load on the musculoskeletal system, which is important for patients exposed to hypokinesia
• High resistance for performing movements
• Gait and posture improvement
• Limitation of joint and ligament hypermobility
• Sole compression to counteract its pathological disposition
• Increase in intensity of proprioceptive input
• Ability to develop personalized treatment plan according to patient’s movement disorders, due to simplified suit regulation and adjustment (Galanov et al., 2010).

### 3.1 Post-Stroke Rehabilitation

Instability and imbalance are serious problems for patients with cerebral stroke. Even in the absence of paresis, patients who had a vertebrobasilar stroke often suffer from pronounced impairments in the function of movement, which limit their life and social activity. There is an increased risk of sudden falls of patients with a history of cerebral stroke: about a fifth of them falls during subsequent 2–2.5 years following a stroke, and up to a half of such falls can result in serious injuries (Lim et al., 2012). There are sufficiently valid tools for risk assessment of potential falls, for example, Berg Balance Scale (Tilson et al., 2012). A more objective approach involves the use of stabilometric equipment. 40 patients (21 women and 19 men, mean age 61 ± 4 years) with ischemic stroke in the vertebrobasilar area in the early recovery period (21 days–6 months post stroke), underwent a complex technique of balance and stability disorders assessment, including the use of clinical rating scales and stabilometric examination. Compared to the assessment scheme commonly used in clinical practice, which usually includes only non-instrumental approaches, the addition of stabilometric criteria improves the diagnosis of existing disorders and makes it possible to objectively monitor the condition of patients during treatment and rehabilitation. All patients received treatment in accordance with the existing standards. At the same time, 20 patients out of 40 (13 women and seven men, mean age 62 ± 8 years) received additional therapy, including a set of rehabilitation exercises (vestibular and respiratory gymnastics), support reaction biofeedback (based on stabilography), training on a stabilometric platform in an axial loading suit “Regent”. The control group (20 patients) was comparable to the main group in all parameters. At the beginning of treatment, average score on the Berg Balance Scale for all patients was 38 points, at the end it was 42 points. In five patients out of 40, the Berg Balance score did not change. It should be noted, that an increase in dynamics for this scale was better observed in those 20 patients who received additional treatment–Berg Balance Scale average score increased from 38 to 46 (by ∼19%), and among the remaining 20 patients without additional treatment, it increased from 37 to 39 (by ∼5%) (Romanova et al., 2014).

In another study, 28 patients with acute stroke in the vertebrobasilar area, suffering from dizziness, equilibrium and stability disturbances, were randomized into two equal groups (*n* = 14). The control group underwent traditional pharmacological and physical treatment, while the main group was additionally treated with “Regent” suit. Rehabilitation of patients in acute period of ischemic stroke with “Regent” suit significantly improved their stabilometric parameters (the Romberg’s test) and vertical stability assessed with various clinical scales (Bohannon, Perry, Stolyarova, Berg Balance Scale). According to the Berg Balance Scale, vertical stability of the main group patients was 40% (р<0.001) higher than in the control group at the end of treatment (Kubriak et al., 2014).

Russian Research Center of Neurology also performed a study investigating the effectiveness of “Regent” axial loading suit in rehabilitation of patients with focal lesions of central nervous system (consequences of acute cerebrovascular disturbances, traumatic brain injuries). 324 patients (197 male and 127 female) with ischemic cerebrovascular pathologies were involved in this study. The control group (100 patients) underwent traditional complex rehabilitative treatment; the main group complex treatment also involved training with “Regent” suit. After the course of rehabilitation there was a more significant decrease in movement disturbances in the main group compared to the control group. Degree of paresis decreased in both groups, however, this change was more significant in the main group (by 33%), than in the control group (by 20%). Muscle spasticity also decreased in both groups: by 18% in the main group and by 15% in the control group. State of bathyesthesia increased in comparison with pre-rehabilitation tests (by 31% in the main group and by 22% in the control group). Average walking speed also increased to a higher extent in the main group (by 33%), than in the control group (by 17%). It is interesting to mention, that the use of “Regent” suit had a positive effect not only on the patients’ locomotor patterns, but it also improved their neuropsychological functions (pronunciation and grammar skills correction, active vocabulary enlargement, etc.) (Chernikova, 2016).

### 3.2 Rehabilitation of Children With Cerebral Palsy

Cerebral spastic infantile paralysis (cerebral palsy, CP) is one of the most common childhood disabilities (Vitrikas, Dalton and Grant, 2020). According to worldwide statistics, cerebral palsy occurs in 2.5 children out of 1000 (Mavlyanova, 2020). There are various clinical features of CP, encompassing a broad range of abnormalities, which mostly include non-progressive movement disorders, poor balance and sensory deficits (Vitrikas, Dalton and Grant, 2020). Different treatment strategies, including physiotherapy, occupational therapy and the use of orthoses are widely applied in mitigation of disabilities, provoked by CP. Along with that, in the last decade, anti-spasticity drugs and orthopaedic surgery were used. The most up-to-date treatment and rehabilitation approach most likely involves the use of therapy garments and orthoses (Romeo et al., 2018). The existing therapy garments include “Adeli” suit, “TheraSuit”, full body suit (Kendall-Camp United Kingdom Ltd.), dynamic elastomeric fabric orthosis (DEFO), stabilizing pressure input orthosis (SPIO), “UpSuit”,United Kingdom “Second Skin” and “PediaSuit” (Karadağ-Saygı and Giray, 2019).

A single-subject report investigating long-term outcomes of “Adeli” suit treatment (AST) described its high efficiency in improving gait, gross motor function and balance in a child (female, 8 years old) with diplegic cerebral palsy. AST was applied for a total of 50 min, once a week, for 18 weeks. In a 10-m walking speed test, the time of test performance significantly decreased (*p* = 0.014) after AST. It is important to mention, that a notable decrease in test performance time was already observed after the very first AST session (Ko et al., 2015). However, AST usually consists of lengthy treatment sessions, which are more frequent, but the overall treatment program is shorter: they mostly last 5–6 h a day, 6 days a week, for 4–5 weeks (Turner, 2006). In another study, 24 children with CP were treated for 4 weeks (2 h daily, 5 days per week, 20 sessions). The aim of this study was to investigate whether children with CP receiving physical therapy with AST would better improve their motor function and mechanical efficiency than children receiving regular therapy based on neurodevelopmental approach (NDT). Children were matched by age and functional status and randomly assigned in two groups: AST group (*n* = 12; eight males, four females; mean age 8.3years) and NDT group (*n* = 12; nine males, three females; mean age 8.1year). The study specifically revolved around the effects of AST on gross motor function and energy cost quantified by mechanical efficiency. There was a trend for reduction in metabolic cost for a given amount of external work after AST compared with NDT. A significant time effect for gross motor function in all participants after 1 month of intensive physiotherapy treatment was greater than expected from the usually observed in children with CP at that age. These findings support the idea that intensive therapy, either AST or NDT can generally accelerate the acquisition of motor abilities in children with CP. The study also showed that AST can optimize motor skills in children with initially higher level of gross motor skills, as reflected by a reduced metabolic cost of external work (Bar-Haim et al., 2006). Another study of AST approach by K.A. Semenova (1997) reported on 60 children with spastic diplegia and 34 children with hyperkinetic diplegia who underwent AST and traditional treatment. Major improvements were reported with AST approach in comparison with the traditional treatment (protocol not specified), but statistical methods and analysis were unclear (Semenova, 1997).

## 4 Mechanical Stimulation of the Soles’ Support Zones for Neurorehabilitation

**FIGURE 2:**
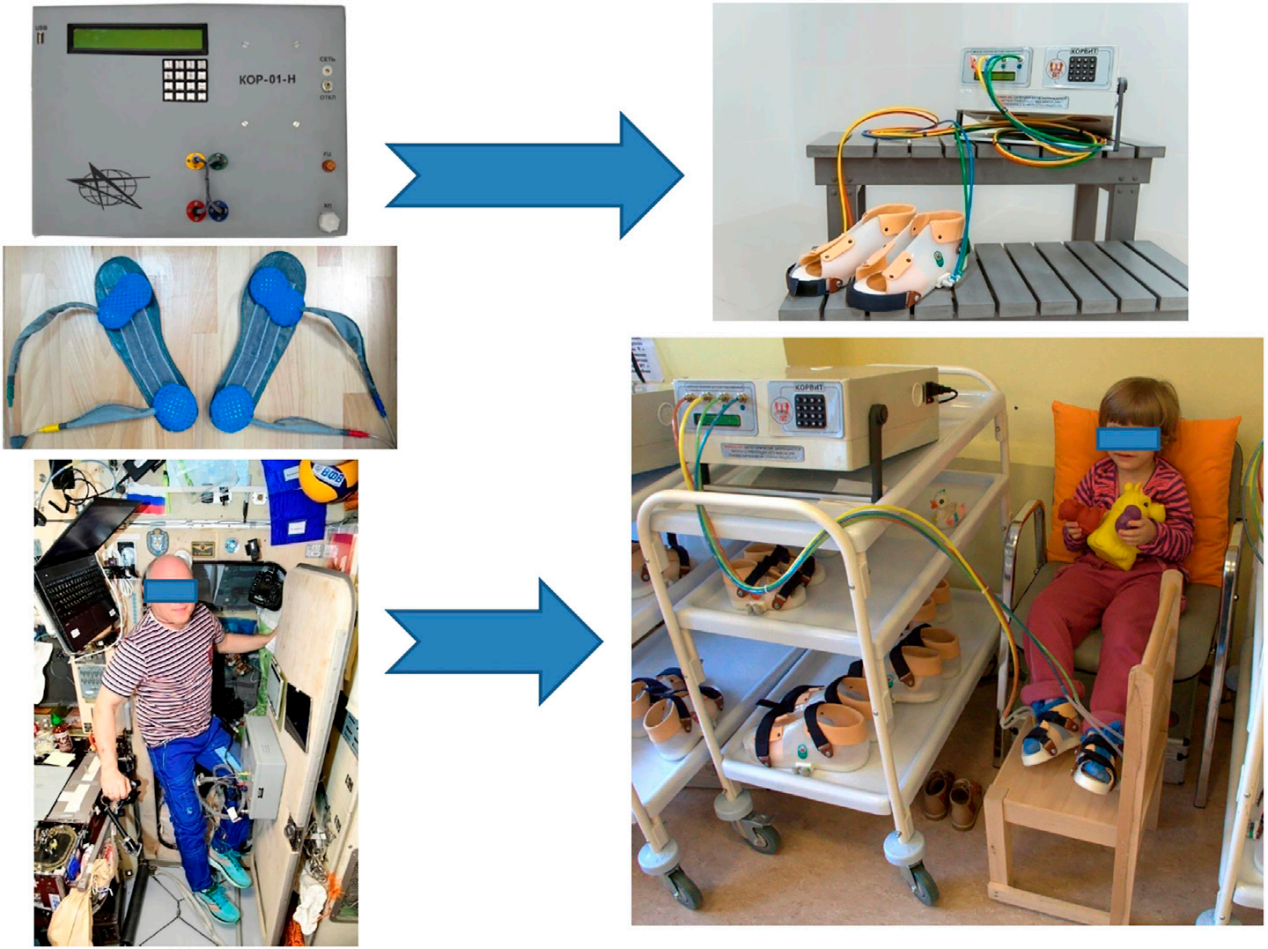
Compensator of support unloading. On the left—space device “KOR” and its application on board the International Space Station; on the right–its clinical version “KORVIT” (Center of Aviaspace Medicine and Technologies, Russia) used for rehabilitation of patients. Photo credit IBMP.

In order to mitigate weightlessness-derived movement disorders, scientists at the Institute of Biomedical Problems of the Russian Academy of Sciences together with the VIT company (Russia) developed another unique device “Korvit”,United Kingdom a plantar compensator of support unloading (Figure 2). “Korvit” allows to reproduce the support stimuli inflow that occur in the process of natural locomotion. The device consists of a control unit, a power supply module, MRI-compatible air ducts, and orthoses with bladders inbuilt into soles that are fixed on the subject’s feet. “Korvit” creates pneumo-mechanical pressure on the foot support zones using bladders that operate in real locomotion regimes (75 steps per minute, 40 kPa pressure). These bladders are inbuilt in such a way as to stimulate the feet areas with maximum density of support mechanoreceptors (Fater-Paccini and Meisners’ bodies) (Kremneva et al., 2013). Another important “Korvit” feature is that it allows to perform rehabilitation regardless of the patient’s immobility degree (Chernikova et al., 2013).

### 4.1 Rehabilitation of Children With Cerebral Palsy

Proprioceptive stimulation is one of the methods with proven effectiveness in improving motor activity. In children with motor disorders, proprioceptive impulses can normalize to a certain extent the activity of damaged nervous system structures that control motility. It can also slow down and prevent the development of pathological changes in the musculoskeletal system (Prityko et al., 2019). The method has been tested in a study of 87 children (28 girls and 59 boys) with various forms of cerebral palsy aged from 1 to 16 years. Сlinical examination of children with spastic diplegia after the course of support stimulation showed a decrease in the severity of tonic reflexes and a decrease in muscle tone, which manifested in positive dynamics of spasticity tests, full foot support acquisition, and absence of leg scissoring. Eight children under the age of two (53%) developed new motor skills: the ability to independently sit down from a supine position, leaning on one arm; to stand and move with walking aid. Five children under the age of four (14.7%) began to walk independently. Children who originally were able to move independently changed the pathological stereotype of walking due to the extinction of primitive tonic reflexes: hip extension increased; the internal rotation of hips and the pathological feet position decreased; foot rolling pattern appeared (rock-up from heel to toe). In patients with the hemiparetic form of cerebral palsy, manifestations of paresis decreased, while the range of motion in the ankle and knee joints of the paretic limb increased. Children could walk a greater distance without visible fatigue, fully loading both feet. In children with the atonic-astatic form of the disease, muscle tone increased, muscle strength and stability increased when walking, coordination of movements improved, manifestations of motor ataxia and motor awkwardness decreased. These results indicate that support stimulation with “Korvit” is a pathogenetically justified, effective and safe method of rehabilitation treatment of children with cerebral palsy. (Levchenkova et al., 2012).

“Korvit” is also successfully used for effective rehabilitation of patients after traumatic brain injuries which caused various movement disorders (Petrova et al., 2019), of patients with Guillain–Barré syndrome (acute sensory-motor polyneuropathy) (Khoroshun, Piradov and Chernikova, 2012) and of children with shin fractures (Serova, Tishchenko and Nikishov, 2012).

### 4.2 Post-Stroke Rehabilitation

Depending on the stroke stage (acute, sub-acute or chronic), rehabilitation therapists use various techniques and methods to restore patients’ damaged functions (Shvarkov et al., 2011; Nordin, Xie and Wünsche, 2014). Rehabilitation measures in acute and sub-acute stroke stages are aimed at reducing functional deficiency and prevention of post-stroke complications. First of all, passive kinesiotherapy (passive movements in all large joints) is performed. It allows to activate the flow of afferent impulses to the perifocal zone of functional asynapsia in the brain, and switch on the motor zones of the cerebral cortex. Another treatment method is verticalization: either passive (with the assistance of verticalizer table), or active. The purpose of passive verticalization is to perform orthostatic training, to preserve afferentation from articular and muscle-tendon receptors and prevent thrombosis in the veins of lower extremities. During this time period, rehabilitation therapists begin proprioceptive stimulation of the soles’ support zones with “Korvit” (Shvarkov et al., 2011).

The scientists of Russian Research Center of Neurology proved the clinical effectiveness of the soles’ support zones mechanical stimulation. They showed that it normalizes muscle tone in the paretic leg and prevents the development of severe spasticity in the extensors of the foot, as well as contributes to an earlier stance and independent movement skills development. The study included 45 patients aged 61 (55.0; 66.0) years with an average of 1 day (1.0; 2.0) from the onset of moderate and severe stroke. The study group was comprised by 24 patients who received both mechanical stimulation of the plantar support with “Korvit” device (75 steps per minute) and conventional therapy in the first hours of stroke. The control group consisted of 21 patients who received only conventional therapy. All patients on admission and 21 days post-stroke were tested according to international clinical scales (NIHSS, Rankin, Barthel, Fugl-Meyer, Ashworth). It turned out that the inclusion of “Korvit” stimulation in the complex of rehabilitation measures can accelerate the restoration of muscle strength in the paretic leg, improve balance in the sitting and standing positions, and walking. In addition to that, the inclusion of “Korvit” in the early post-stroke rehabilitation program (from the very first day of rehabilitation) improves muscle tone in paralyzed limbs. It can be suggested that “Korvit” stimulation is effective at early stages of rehabilitation after acute stroke primarily due to correction of postural and tonic disorders triggered by functional support deprivation in the gravitational muscles (Glebova, Maksimova and Chernikova, 2014). The obtained results can be explained on the basis of the study by Tomilovskaya Elena (Tomilovskaya et al., 2013), who showed that the use of mechanical stimulation of the soles’ support zones performed in the mode of locomotion leads to activation of spinal locomotor structures. In addition, clinical neuroimaging studies, performed in the Russian Research Center of Neurology showed that mechanical stimulation of the soles’ support zones in standing and slow walking modes provokes activation of supraspinal structures involved in control of locomotion (primary soma of the sensory cortex, premotor, dorsolateral prefrontal cortex and insular lobules). Simulation of standing was accompanied by greater involvement of the prefrontal cortex. Simulation of slow walking mostly involved sensorimotor parts of the cortex and triggered motor synergies (Kremneva et al., 2012; Glebova, Maksimova and Chernikova, 2014).

## 5 Complex Rehabilitation Therapy

Nowadays, some clinicians use complex rehabilitation therapy, which includes both: axial loading suit and soles’ support stimulation. Such complex approach was assessed in a 15-year study of 3000 post-stroke patients aged from 20 to 86 years. They were divided into several pathology-dependent groups:

1 80%—rehabilitating from the consequences of acute violations of cerebral circulation in the early and late recovery periods;
2 14%—rehabilitating from the consequences of severe traumatic brain injury;
3 4%—patients rehabilitating after neurosurgical brain surgery;
4 2%—rehabilitating from spinal strokes and their consequences.

The duration of rehabilitation treatment averaged 21 days. The main clinical manifestation in all groups was deep spastic hemiparesis. Neurorehabilitation activities included individual sessions with rehabilitation therapist, who, in addition to performing massage and exercise therapy, also used axial loading suits and proprioceptive stimulation with “Korvit” device. Clinically significant improvement after the treatment was observed in 61% of patients. Improvement of occupational skills was achieved in 30% of patients, 19% of which returned to their previous work. Recovery of walking skills and everyday life self-care improved in 70% of patients. Walking stereotype improved in 29% of patients. 28% of patients began to walk independently, and in 24% of patients the number of stops due to muscle weakness decreased. There was a significant (*p* < 0.05) increase in muscle strength and a decline in muscle tone in all groups. Therapeutic effects also included a decrease in the degree of upper limbs paresis, an increase in pace frequency and movement volume, and correction of movement coordination. Patients also noted an improvement in nocturnal sleep, overall condition and mood (Shvarkov et al., 2011).

Complex and personalized application of the above listed methods in neurorehabilitation programs makes it possible to effectively treat neurological patients even with significant brain damage by activating plastic processes in the brain. The results obtained by the Russian Research Center of Neurology are already actively used in clinical practice, and in the near future may be included in the rehabilitation programs of Russian clinics and hospitals specializing in restorative medicine (Piradov, Chernikova and Suponeva, 2018).

## 6 Conclusion

Summing up, it can be said that space technologies have made a great contribution not only to space medicine, but they have strongly influenced the development of terrestrial medicine, which actively involves these technologies in everyday practice. Methods of proprioceptive correction are aimed at complex restoration of neurological functions, thus, increasing the effectiveness of rehabilitation treatment of patients suffering from severe motor disturbances, triggered by children cerebral palsy, ischemic stroke, traumatic brain injuries and other spinal or cardiovascular pathologies. Their application helps to recover disturbed motor stereotype and prevent maladjustment to everyday life. In addition to that, inclusion of these means in early rehabilitation programs enhances the rehabilitation process and makes it more effective. Complex application of proprioceptive correction methods in neurorehabilitation programs makes it possible to effectively treat neurological patients even with significant brain damage. However, it is clear that these methods should only be applied as additional therapy along with conventional rehabilitation techniques. Their isolated administration would most likely be ineffective, especially in patients with severely damaged motor functions. The severity of motor disturbances is also a limiting factor for application of axial loading suit, as it cannot be used for patients who are paraplegic.
